# The Interaction of Morphological and Stereotypical Gender Information in Russian

**DOI:** 10.3389/fpsyg.2015.01720

**Published:** 2015-11-16

**Authors:** Alan Garnham, Yuri Yakovlev

**Affiliations:** School of Psychology, University of SussexBrighton, UK

**Keywords:** gender, stereotype, grammatical gender, text comprehension, Russian

## Abstract

Previous research, for example in English, French, German, and Spanish, has investigated the interplay between grammatical gender information and stereotype gender information (e.g., that secretaries are usually female, in many cultures), in the interpretation of both singular noun phrases (*the secretary*) and plural nouns phrases, particularly so-called generic masculines—nouns that have masculine grammatical gender but that should be able to refer to both groups of men and mixed groups of men and women. Since the studies have been conducted in cultures with broadly similar stereotypes, the effects generally reflect differences in the grammatical systems of the languages. Russian has a more complex grammatical gender system than the languages previously studied, and, unlike those languages frequently presents examples in which grammatical gender is marked on the predicate (in an inflection on the verb). In this study we collected stereotype norms for 160 role names in Russian, providing a useful resource for further work in this language. We also conducted a reading time study examining the interaction of grammatical and stereotype gender information in the interpretation of both Russian singular noun phrases, and plurals that were (potentially) generic masculines. Our results show that, although both types of gender information are used, when available, the effects of grammatical marking on the predicate are not as strong as those of such marking on subject noun phrases.

## Introduction

The understanding of written text and spoken discourse depends on the integration of information that is explicitly presented in the text with background information, both specific and general, that the comprehender has. The combination of these two types of information should be straightforward according to theories, such as the theory of mental models (Johnson-Laird, 1983), that claim the two types of information have the same format. The combining of the two types of information can produce additional pieces of information, which, if the information is in the form of descriptions of part of real or imaginary worlds, can be regarded as inferences from what is explicit in the text, making use of background information. There has been some debate about the extent to which inference making occurs routinely during comprehension. McKoon and Ratcliff's (1992) minimalist hypothesis, for example, claim that inference making is relatively restricted. Alternative, constructivist accounts (e.g., Graesser et al., 1994) place more emphasis on inference making, attempting, for example, to identify classes of inference that are routinely made, or circumstances in which inferences are made in an “effort after meaning.” To some extent the minimalist vs. constructivist dichotomy is a false one (Garnham, 1992), and it may be more productive, or even necessary, to pursue a common theme in both approaches, which is to specify and investigate factors that determine when inferences are made.

One idea that we have pursued (e.g., Garnham, 2005: 248) is that inferences that depend strongly on the presence of particular lexical items in a text might be particularly easy to make, as the template for the inference is (very likely) retrieved as the lexical item is processed. One domain in which we have investigated this idea is that of social stereotypes associated with occupational and other social roles. We would not claim that our work on stereotypes has entirely supported our ideas. There are, for example, terms (in British English) such as *primary school teacher* that would not typically be thought of as single lexical items, but that behave very similarly, from a gender stereotyping point of view, to lexical items such as *secretary*. The original idea behind our work, which was primarily a study of inferencing rather than a study of stereotypes, was that a word such as *secretary* would be directly linked in memory to the knowledge that, in the society in which our experimental participants moved, a high proportion of secretaries are female. So, in interpreting a statement presenting a particular individual as *the secretary*, this information would probably come to mind, and could result in the individual being represented as either probably a female or even definitely a female (though in a way that could be overridden if later information indicated that the person was male).

Of course, there is a sense in which such inferences should not be made. The core meaning of a word such as *secretary* is the information that defines that role. And it is a role that can be fulfilled by either females or males. From that point of view, simply describing someone as *the secretary* says nothing about their gender. Nevertheless, we found clear evidence that, in reading simple English texts, people do take secretaries to be (probably) female and engineers to be (probably) male (e.g., Carreiras et al., 1996; Garnham et al., 2002; Oakhill et al., 2005). Some of our individual findings do not clearly show that this inference is made as the word *secretary* or *engineer* is read, as the effect shows up when a later coreferential pronoun *he* or *she* occurs. Nevertheless, we believe that our results overall do support the idea of an immediate inference (see particularly the arguments in Reynolds et al., 2006).

As an Indo-European language, English is relatively unusual in that its nouns do not fall into grammatical gender categories and, except for pronouns and certain derivational endings, such as –ess, which are falling out of favor, there is little morphological marking for gender, for example on articles and other determiners, nouns, and adjectives. So, in our English experiments we would typically have a noun phrase, such as *the secretary* or *the engineer* that was not grammatically marked for gender followed by a pronoun *he* or *she* or *him* or *her* that was. At the initial noun phrase, therefore, stereotype information had free reign to determine the representation of the sex and/or gender of the protangonist. In other European languages that we have studied, such as Spanish, French, and German, the situation is different. Determiners are inflected (for example, Spanish: *el/la*; French: *le/la* German: *der/die/das*), and there may also be indications in the noun ending of likely gender (for example, Spanish: *-o/-a*). Thus, grammatical and stereotype information may be working either together or against each other to provide information about the sex/gender of a person talked about in a text. Indeed, our research (Carreiras et al., 1996; Gygax et al., 2008) shows different patterns of results for each of the languages studied, which can be explained by the interaction of their different patterns of morphological marking of gender and the relatively constant effects of gender stereotyping. A further complication in these languages, which we have studied in the context of French and German, is the generic use of the masculine. A particularly pertinent case, and the one we capitalized on (Gygax et al., 2008), is when a speaker or writer is not sure or does not wish to commit to whether a group of people is all male, all female, or mixed. In such cases, a masculine plural noun phrase can be used (e.g., French: les assistants sociaux; German: Die Sozialarbeiter; English: the social workers). However, at least locally, the use of such an expression will be ambiguous between this generic case and cases in which the composition of the group is known to the entirely male. A difference between German and French, which appeared to influence our results, is that the German masculine plural definite article has the same spelling as the feminine singular *die*.

There is also some evidence from Russian on the interpretation of generic masculine nouns. Doleschal (1993) conducted an experiment in which she investigated how generic masculine nouns denoting persons are interpreted in Russian. The results of the study showed that, when attention shifts from the speaker's to hearer's perspective, neutral-masculine nouns are predominantly perceived and interpreted as male. However, it has been noted that some masculine nouns, such as *bukhgalter*^1^ (accountant) and *vrach* (doctor) received higher female scores than male. Doleschal's assumption was that there might be an influence of what she called extra-linguistic factors, knowledge that some occupations are typically female. Moreover, some of the common gender nouns, for example *nedotroga* (touchy person) and *umnitsa* (know-all), also were interpreted as female. Similar results were obtained by Schmid (1998, cited in Doleschal and Schmid, 2001: 266), whose data suggested a strong tendency for interpreting masculine-neutral (generic) nouns as denoting men, though with some exceptions.

In the present study, we further investigate these issues in Russian. In the first part of the study we collect a new and more comprehensive set of stereotype norms for 160 Russian role names. The second part is an experimental study of stereotyping in short Russian texts, using the self-paced reading technique that has been used in previous studies. More specifically, we investigated the interpretation of masculine nouns with either masculine, neutral, or feminine stereotypes, and how the stereotype information interacted with grammatical information, in particular the inflection on the main verb in the sentence containing the stereotyped role name, and the gender of a definite pronoun in the following sentence. We looked at role names in both singular NPs, which we intended to be interpreted as referring to a specific person, and plural NPs, which were intended to be interpreted generically. The nouns fell into three different groups, with different gender-related properties, and we were interested in whether nouns from the three groups behaved differently. These groups of nouns are described in detail below. Before describing the studies, we present some information about grammatical gender in Russian, which is more complex than in the other languages we have studied, and can be hypothesized to have different effects on processing.

### Grammatical gender in russian

Russian is a language in which gender is predictable from semantic and morphological factors. Russian nouns are divided into three gender classes: feminine, neuter, and masculine (though see below for the notion of a fourth, common, gender). All nouns are marked grammatically and agree with adjectives, verbs (past-tense) and pronouns, the form of which depends on the gender of the noun they refer to. Gender agreement with the verb is a major difference between Russian, on the one hand, and English, French, German, and Spanish, on the other. An important question, therefore, will be whether gender marking on the verb acts in the same way, in on-line processing, as the types of gender marking studied in English, French, German, and Spanish (on determiners, nouns, and pronouns).

Russian also contains declension classes for nouns, and there is a strong relationship between gender and declension class. Traditionally nouns are categorized into three major classes:

- Declension I

Nouns in declension I are mainly feminine, though some are masculine. In the nominative singular they end in –*a* or –*ya*:

*glina*—clay (f), *zemlya*—earth (f), *yunosha*—youngster (m), babushka—grandmother (f).

- Declension II

Nouns in declension II are mainly neuter, though a few are masculine and neutral nouns. In the nominative singular they either end in a consonant or in -*e* or -*o*:

*vecher*—evening(m), *utro*—morning (n), *zadaniye*—task(n), *bereg*—coast(m).

- Declension III

Nouns in declension III are feminine. In the nominative singular they end in a soft consonant:

*noch'*—night (f), *rol*′—role (f)

Noun agree in gender with adjectives, participles (in the singular), and verbs (in the past tense), as illustrated in the following examples:

*nastal teplyy vecher*—a warm(m) evening(m) came(m)
*nastala teplaya noch*′—a warm(f) day(f) came (f)
*nastalo teploye utro*—a warm(n) morning(n) came (n)

Each gender category has an animate and inanimate subgender. For inanimate nouns the assignment of grammatical gender is (semantically) arbitrary, as in other gender-marked languages. For example, sun is neutral in Russian (*solntse*), feminine in German (*die Sonne*) and masculine in French (*le solei*) and Spanish (*el sol*). However, grammatical gender correlates strongly with morphological factors, and in particular declension class (Corbett, 1982, 1991). All nouns denoting human beings are animate and normally belong to either masculine or feminine grammatical gender, depending on their semantic gender. Only a few animate nouns have neutral gender, for example *chudovishche* (monster) and *zhivotnoye* (beast). Nouns that denote people are known as personal nouns. They can be divided into six classes or groups:

a. Paired nouns with independent words for each gender.
  medsestra—medbrat
    nurse (f, sg.)—nurse (m, sg.)/(medical sister—medical brother)
  evropevka—evropevets—evropevki—evropevtsy
    European (f, sg.)—European (m, sg.)—Europeans (f, pl.)—
    Europeans (m, pl., generic)

b. Paired nouns with masculine nouns that can denote a female person in contexts when gender is not important.
  studentka—student—studentki—studenti
    ‘student (f, sg.)—student (m, sg.)—students (f, pl.)—
    students (m, pl., generic)’
  *uchitel*′*nitsa - uchitel*′*- uchitel*′*nitsy—uchitelya*
    ‘teacher(f.sg)—teacher(m.sg)—teacher(f. pl.)—teachers (m.pl. generic)’

c. Masculine nouns that do not have a feminine counterpart.
  kosmetolog—kosmetologi
    ‘beautician (m., sing)—beauticians (m, pl., generic)’
  electrik—electriki
    ‘electrician (m, sg.)—electricians (m, pl., generic)’

d. Masculine nouns that have only a so-called “colloquial” feminine counterpart. The use of colloquial feminine nouns goes against the norms of modern literary Russian (and they usually carry a negative connotation, if used). In addition, their use is ambiguous as they were used in the past to refer to a female person who was married, for example, to a doctor or a professor.
  vrachikha—vrach—vrachi—vrachikhi
    ‘doctor (f. sg. colloq.)—doctor (m. sg.)—doctors (m. pl., generic)—doctors (f. pl. colloq)’
  *parikmakhersha—parikmakher—parikmakhery*—*parikmakhershi*
    ‘hairdresser (f. sg. colloq.)—hairdresser (m. sg.)—hairdressers (m, pl., generic)—hairdressers (f. pl. colloq.)’

e. Feminine nouns that do not have male counterpart. In contrast to masculine personal nouns that do not have feminine counterpart, they cannot denote a male person. The only exceptions are in metaphorical expressions.
  balerina—baleriny
    ‘ballerina (f, sg.)—ballerinas (f, pl.)’
  nyanya—nyani
    ‘nanny (f, sg.)—nannies (f, pl.)’

We note however, that it is possible to create masculine counterparts for these nouns (e.g., *baleron/balerun—*male ballet dancer or *nyan'—*male nanny), but their use would be colloquial and almost always humorous. The appearance and popularization of *nyan'* is related to relatively recent release of a comedy film “The Sitter” (2011). The word *“balerun”* was popularized in 90s after several ironical usages in the press.

f. Common gender nouns (mostly in spoken language) denote both males and females. This gender is different from the neutral gender. Common gender nouns all end in *-à/-ya*. So they belong to a declension in which most of the nouns are feminine. However, in terms of modifying adjectives, ordinal numbers, pronouns, and past tense verbs, agreement depends on the semantic gender of the individual in question.
  plaksa—plaksy
    ‘weeper (common gender, sg.)—weepers (common gender, pl.)’
  kollega—kollegi
    ‘colleague (common gender, sg.)—colleagues (common gender, pl.)’

The gender of a personal noun can be unambiguously determined on the basis of its semantic or morphological agreement with other syntactically dependent words (e.g., adjectives and pronouns). However, semantic agreement is complicated by the existence of classes c, d, and f, described above. For example, masculine nouns that do not have a feminine counterpart may trigger feminine agreement in certain syntactic positions (1):

(1) *kosmetolog skazala*
      ‘the beautician (m) said (f)’

Corbett (1991) argued that the agreement of these nouns is subject to an agreement hierarchy (attributive—predicate—relative pronoun—personal pronoun), with semantic agreement becoming increasingly common from left to right. So, (2) is relatively acceptable, with sematic agreement in the predicate, but (3) with attributive semantic agreement is not.

(2) *budushchiv filolog skazala*
      ‘future (m) philologist (m) said (f)’

(3) ya vstretil tvoyu kosmetologa^*^ (not allowed)
      ‘I met (m) your (f. accusative) beautician (m. accusative)’

In contrast, common gender nouns have two consistent agreement patterns (feminine and masculine). The choice of gender depends only on the semantic gender of the referent. See example (4) below.

(4) *nash novvy kollega skazal*
      ‘our (m) new (m) colleague (common gender) said (m)’
  nasha novaya kollega skazala
      ‘our (f) new (f) colleague (common gender) said (f)’

In cases where the gender of a person is unknown or irrelevant, Russian uses masculine forms, which are seen as stylistically neutral. For example, in official contexts it is more appropriate to use “masculine-neutral” nouns. In plural forms masculine nouns are regularly used generically.

## Rating study

The aim of the rating study was to produce norms for the gender stereotypicality of selected role nouns in Russian. The norms are of interest in themselves, but are also needed to construct items for the main on-line experiment, because appropriate norms for Russian do not exist. Of the six classes of personal nouns described above, only three (b, c, d) were included in rating study: masculine nouns with feminine counterpart (“paired”); masculine nouns with colloquial feminine pair (“colloquial”); masculine nouns without a feminine counterpart (“unpaired”)^2^. We looked at whether nouns in the different groups received significantly different ratings.

Ethical approval for this study and for the online study that follows was granted by the University of Sussex Life Sciences & Psychology Cluster-based Research Ethics Committee and all participants provided written consent prior to taking part. All procedures complied with the British Psychological Society's Code of Human Research Ethics.

### Methods

#### Questionnaire and design

Gender stereotypes for 160 role nouns in Russian were evaluated in an online questionnaire. The selection of role names and the design of the survey were based on previous studies in other European languages (Kennison and Trofe, 2003; Gabriel et al., 2008; Misersky et al., 2014). The role nouns were divided into three groups: gender paired nouns, where the masculine noun can refer both to men and women (*n* = 44); masculine nouns without a feminine counterpart (*n* = 55); masculine nouns which have a feminine counterpart, but only one that is used colloquially (*n* = 61).

Role names were presented in the masculine plural form (serving as generic) on the left side of the screen, slightly separated from an 11-point rating scale, which ranged from 100% women and 0% men on the left to 0% women and 100% men on the right. Previous work (Gabriel et al., 2008) suggests that presenting generic masculine forms of the role names, as opposed to explicitly gender marked versions, can increase the perceived proportion of males. However, we decided not to use specific feminine and masculine personal nouns because of the inclusion of nouns with colloquial feminine counterparts. Some of these feminine forms are archaic, some vulgar, and they are rarely, if ever, used in written form. As in other similar studies (Gabriel et al., 2008), our data showed that some participants interpreted the masculine generic version of some nouns (e.g., florists) as specifically male despite the fact that they were embedded in the series of personal nouns that were generically interpreted. However, the comparability of our results with previous results in other languages (e.g., Misersky et al., 2014) suggests that such responses did not constitute a serious problem.

Another issue is that ratings are influenced by scale direction (Kennison and Trofe, 2003; Gabriel et al., 2008). A scale with 100% male on the right is associated with a numerically small but significant increase in the tendency to rate nouns as referring to males. Given that the effect is small, we decided to use one scale direction only.

After reading the instructions and indicating their consent form, participants were asked to estimate the proportion of females vs. males in each role. The list of the nouns was randomized and presented in the same order to all participants. On the last page of the questionnaire participants were asked to indicate their native language and answer optional demographical questions (age group, gender, education level). The questionnaire was created using Bristol Online Surveys (BOS) and administered via the Web. Its design is demonstrated in Appendix A (Supplementary Material). The list of nouns is given in Appendix B (Supplementary Material), together with summary data from the survey for each noun.

#### Sample and procedure

A total of 112 participants took part in the rating study. They were recruited via advertising in Russian social networks, and participation was on a voluntary basis. Data from six participants were excluded from the analysis because Russian was not their mother tongue (*n* = 4) or because they did not understand the instructions (*n* = 2). The final sample, therefore, consisted of 106 participants (16 male, 87 female and 3 who chose not to specify their gender).

### Results

Data from the questionnaire was coded so that high values on the scale reflect a higher proportion of men, for example “100% women and 0% men” was recorded as 1, “50% women and 50% men” as 6 and “0% women and 100% men” as 11.

#### Interparticipant analyses

For each participant, the mean rating across the role names was calculated (*M* = 6.38, *SD* = 0.33, range 5.26–7.34; scale midpoint = 6). The overall distribution of scores was normal, Kolmogorov–Smirnov's *D*_(106)_ = 0.06, *p* = 0.2. Female participants rated the proportion of men as being slightly, though not significantly, higher (*M* = 6.4, *SD* = 0.3) than male participants (*M* = 6.3, *SD* = 0.48), [*t*_(101)_ = 1.04, *p* = 0.297]. Overall, the proportion of women and men was rated similarly by participants in four age groups “18–24,” *n* = 24 (*M* = 6.41, *SD* = 0.31); “25–34,” *n* = 43 (*M* = 6.36, *SD* = 0.31); “35–44,” *n* = 25 (*M* = 6.43, *SD* = 0.39); “45–54,” *n* = 13 (*M* = 6.35, *SD* = 0.37), [*F*_(3, 101)_ = 0.32, *p* = 0.82] and at two education level groups “high school,” *n* = 11 (*M* = 6.39, *SD* = 0.27); “university degree,” *n* = 94 (*M* = 6.39, *SD* = 0.34), [*t*_(103)_ = 0.06, *p* = 0.95].

#### Interitem analyses

**Figure 1:**
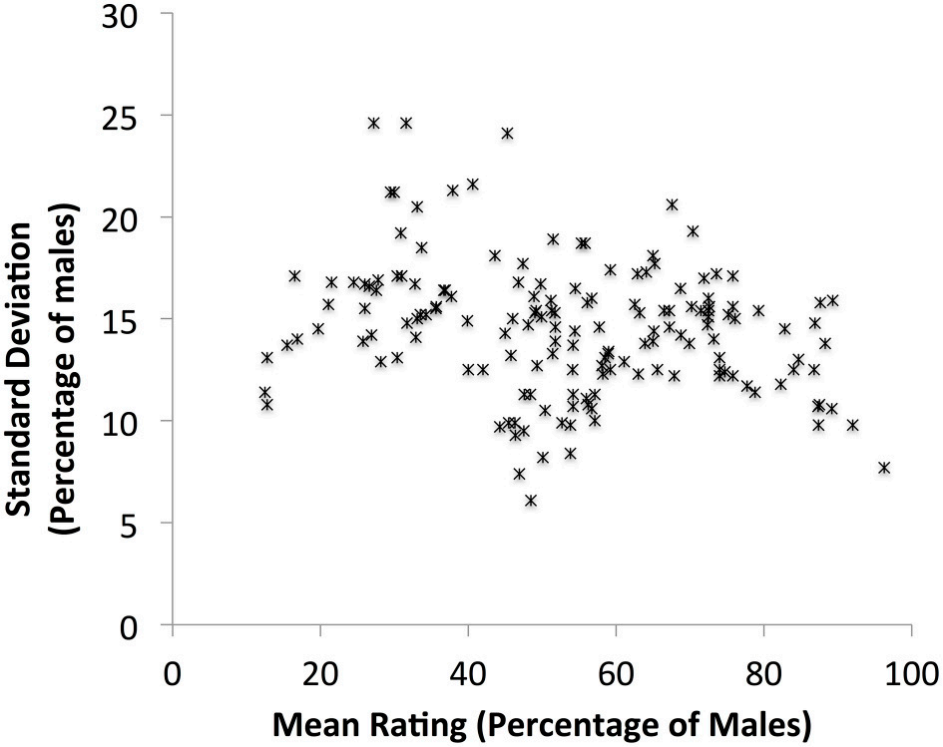
**Scatterplot of mean ratings for each personal noun (***n*** = 160) in the rating study against its standard deviation**.

The mean rating and standard deviation were calculated for each noun (see Appendix B in Supplementary Material for overall data, and for mean ratings by female and by male participants). A scatterplot of the mean rating for each noun against its standard deviation is shown in Figure 1. Low standard deviations, which reflect consensus in perceived proportions, can be seen in the middle and at the both ends of the scale. These results are in line with previous research in English, German and French (Gabriel et al., 2008, Figures 3–5). As in Gabriel et al.'s study more nouns are found at the male than at the female end of the scale.

Means were also calculated for each noun group. Participants rated unpaired masculine personal nouns as denoting a higher proportion of males than females (*M* = 6.78, *SD* = 2.17), paired masculine nouns as denoting close to equal proportions of females and males (*M* = 6.06, *SD* = 1.54), and masculine personal nouns with colloquial feminine pair as denoting a slightly higher proportion of males than females (*M* = 6.25, *SD* = 1.98). A One-way between-items ANOVA showed that the difference between these means is not significant [*F*_(2, 157)_ = 1.92, *p* = 0.15].

## Online experiment

The main experiment investigated the role of gender stereotypes associated with role names on the interpretation of grammatically masculine Russian nouns. Three sets of nouns were selected on the basis of rating study: (a) female stereotyped, (b) neutral, and (c) male stereotyped. The experiment investigated the interpretation of masculine nouns both when they are intended as specific and when they are intended as generic, in passages such as those shown in **Table 3**. Nouns that were intended as specific occurred in the singular form. Nouns that were intended as generically occurred in the plural form. We used passages both in the present tense, where the marking on the verb is not gender-specific, and in the past tense, where it is. We expected to see different results in these two cases.

### Predictions

#### Nouns intended to be interpreted specifically

In the experimental items, the subject noun of the first sentence was always masculine singular, though it could be male, neutral or female stereotyped, and it might turn out to refer to a male or a female, depending on the morphological markings in the rest of the passage. The subject pronoun of the second sentence could be masculine or feminine. In addition, in the past tense, the verbs were morphologically marked as masculine or feminine. The markings on both the pronouns and the verbs were determined by the sex of the person being discussed.

The match or mismatch between the grammatical gender of the subject noun of the first sentence and the stereotype was immediately apparent, but any mismatch effect might be mitigated by the use of masculine nouns to refer to females in Russian, both generally, and in the course of this experiment.

The sex of the person referred to, and any clash with other gender information, therefore first becomes apparent at the predicate of the first sentence in the past tense passages and at the subject pronoun in the second sentence in the present tense passages.

Our results in Spanish (Carreiras et al., 1996) suggest that mismatch effects between stereotype and sex of protagonist occur, and can be resolved, at the earliest possible point, predicting a stereotype mismatch effect in the first sentence (only) for past tense passages and in the second sentence (only) for present tense passages. However, as gender marking on verbs has not previously been studied, we cannot be certain it will have the same effect as, for example, the gender marking on the determiners in the Spanish experiment, which is syntactically closer to the noun itself.

Other mismatch effects, in particular between the grammatical gender of the subject noun of the first sentence and the information that determines the sex of the protagonist should also be present, but, for reasons stated above, may not necessarily affect processing. However, we might expect different behavior from the different noun groups. With the paired nouns (i.e., those that have a feminine counterpart), and to a lesser extent with the colloquial nouns (where the feminine counterparts exist, but have different connotations and/or denotations), the use of the masculine form strongly suggests the person being referred to is male. Thus, a feminine-marked predicate in the first sentence (past tense form) is potentially problematic, and may cause processing difficulties. Furthermore, in the present tense passages, the end of the first sentence is reached with no suggestion that the person being referred to is not male. In these passages, a feminine pronoun in the second sentence is likely to be particularly problematic, as the notion that the protagonist is masculine may have become entrenched. A further complication is that such effects may be modified by the stereotype of the role noun in the first sentence. A female stereotyped noun is likely to make reference to a female person more plausible.

#### Nouns intended to be interpreted generically

Gygax et al. (2008) showed that in French and German morphologically marked generically-intended masculine plural nouns were interpreted as referring to groups of males. Given that generic plurals in Russian are also morphologically marked as masculine, a similar effect can be predicted.

### Methods

#### Participants

Twenty volunteers (8 men and 12 women, mean age = 29.9 years, SD = 8.18) took part in the experiment. Most were students and staff from Sussex University and the University of Brighton. They were people from the former Soviet republics of Belarus, the Russian Federation, Ukraine, Kazakhstan, and Latvia, whose mother tongue was Russian. All subjects were entered into a £25 prize draw for their participation, which lasted for about 35 min.

#### Materials

Seventy-two nouns (24 male stereotyped, 24 female stereotyped, and 24 neutral) were selected for the texts with specific nouns. A further 36 nouns (12 male stereotyped, 12 female stereotyped, and 12 neutral) were selected for the texts with generic nouns. The mean stereotype ratings and standard deviations for the selected nouns (on the 11-point scale) are shown in Table 1.

**Table 1:** **Mean ratings and standard deviations for selected stereotyped nouns**.

**Stereotype**	**Specific nouns**	**Generic nouns**
Female	3.8 (0.69)	3.5 (0.69)
Neutral	6.1 (0.29)	6.2 (0.26)
Male	8.3 (0.59)	8.4 (0.43)

The specific nouns were, in addition, taken from three classes (8 paired, 8 unpaired, and 8 colloquial) within each gender-stereotyped group. Table 2 displays the mean ratings and standard deviations for these groups of nouns.

**Table 2:** **Mean ratings and standard deviations for paired, unpaired, and colloquial nouns within each group of stereotyped nouns**.

**Stereotype**	**Unpaired**	**Paired**	**Colloquial**
Female	3.5 (0.79)	4.1 (0.53)	3.8 (0.66)
Neutral	6.2 (0.29)	6.0 (0.31)	6.1 (0.28)
Male	8.4 (0.57)	8.1 (0.72)	8.4 (0.43)

For each noun a two-line text was constructed. For the specific nouns, the noun, which was always grammatically masculine, though stereotypically female, neutral or male, was the subject of the first sentence. The second sentence began with the (Russian equivalent of the English) pronoun *he* or *she*. Each text could be written in either the present or the past tense. In the past tense the predicate in the first sentence was marked for the real-world gender of the character, thus mismatching the grammatical gender of the noun when it referred to a female person (in the present tense the predicate is gender neutral). Each noun was used in all four types of passages: (1) present tense, referential pronoun *she*; (2) present tense, referential pronoun *he*; (3) past tense, referential pronoun *she*; (4) past tense, referential pronoun *he*. The occurrence of a noun in the four types of passage was counterbalanced between participants.

For generic nouns, the first sentence was used to introduce a group of people using a masculine plural role name (female, male, or neutral stereotyped), and the second talked about some of the men or some of the women in the group. Table 3 displays sample sentences.

**Table 3:** **Examples of sample sentences from the reading experiment**.

**Sentence 1**	**Sentence 2**
**SPECIFIC NOUN, PRESENT TENSE PASSAGE**	
*Kosmetolog govorit po telefonu*.	*Ona/On ob”yasnyaet novomu klientu kak ih nayti*.
“The beautician (masc.) is talking on the phone.”	“She/He is explaining to a new client how to find them.”
**SPECIFIC NOUN, PAST TENSE PASSAGE**	
*Kosmetolog govorila/govoril po telefonu*.	*Ona/On ob”yasnyala/ob”yasnal novomu klientu kak ikh nayti*.
“The beautician (masc.) talked (fem./masc.) on the phone.”	“She/He explained (fem./masc.) to a new client how to find them.”
**GENERIC NOUN**	
*Inzhenery stroili model' 3 chasa*.	*Neskol'ko muzhchin/zhenshchin reshili otdokhnut' 15 minut*.
“Engineers (masc., pl., generic) were building the model for 3 hours.”	“Some of the men/women decided to take 15 minutes break.”

After the two sentences were displayed, participants were asked the following question: “Is it possible to use first and second sentences in that way?” Participants were to answer either Yes or No.

To prevent participants from realizing that only masculine nouns were under investigation, there were 20 filler texts that included specific nouns with feminine grammatical gender, and 10 filler texts with feminine plural nouns, which, unlike masculine plurals, cannot be used generically. It was, therefore, possible to construct texts that required a definite No respond to the evaluation question.

#### Procedure

All participants were tested individually in a small, quiet room. Their task was to read each passage at a fast but comfortable speed and to decide whether the two sentences fitted together to make a sensible passage. The experiment was built using the E-Prime 2.0 software (Psychology Software Tools, Pittsburgh, PA)^3^. There were two untimed breaks during the experiment; participants could continue when they were ready by pressing a continue key, the spacebar. The spacebar was also used to advance the presentation of the sentences. The “C” key was used for No responses to evaluation questions and the “M” key was used for Yes responses. Each participant viewed seven practice passages before the main part of the experiment.

#### Experimental design

The experiment investigated two questions in parallel. The first question was about the interpretation of specific nouns followed by pronouns that refer to the male or female person introduced by the noun. We were interested in four factors: (i) Gender stereotype of the noun in sentence 1 (Male, Female, Neutral), (ii) Gender of pronoun in sentence 2 (she vs. he), (iii) class of the noun (paired, unpaired, colloquial), and (iv) Tense of the predicate in sentence 1 (past vs. present; in the past tense the predicate was gender marked, and the gender marking matched that of the pronoun in the second sentence).

The second question was about the interpretation of generic nouns. There were two factors in this part of the study: (i) Gender stereotype of the generic noun in sentence 1 (Male, Female, Neutral); and (ii) continuation in sentence 2 (*some of the men* vs. *some of the women*). For both types of text reading times of each sentence, responses to the classification question, and response times were recorded.

In each case, there were two or four versions of a passage with a particular role noun (masculine vs. feminine pronoun and past vs. present tense for singulars, “some of the men” vs. “some of the women” for plurals). Therefore, four versions of the experiment were created to counterbalance the allocation of items to conditions. A different random order was selected by E-Prime to present the passages to each participant.

### Results and discussion

#### Interpretation of specific nouns

Examination of the histograms for the reading times suggested that 10 s was a sensible cut off to use for both first sentence and second sentence reading times. The lengths of the sentences showed considerable variability: first sentences mean = 51.1 characters, min = 22, max = 105; second sentences mean = 46.4, min = 22, max = 69. Therefore, for the purposes of analysis, raw reading times were replaced with residual reading times, based on regressions of reading time against length in characters (including spaces) calculated separately for each participant and for each sentence (first and second).

Table 4 shows the mean raw reading times, after truncation, for the first sentences of the passages in the past and present tenses. Mixed effects models, with stereotype (female, neutral, male), pronoun (he, she), tense (past, present), and noun group (unpaired, paired, colloquial) as fixed effects were fitted to the residual reading time data using the SPSS MIXED procedure. A compound symmetry covariance structure was chosen for the repeated effects, and random intercepts (only) were selected for both participants and materials, so no issue of covariance structure for random effects arose^4^. Because we expected different effects in the present tense and past tense passages, we also fitted mixed models to these two subsets of the data separately. All reported times are based on the actual set of times, after truncation, in the various conditions of the experiment.

**Table 4:** **Mean reading times (ms) for first sentences for the texts in which the nouns were intended to be interpreted specifically**.

**Stereotype**		**Female**		**Neutral**		**Male**	
**Pronoun in second sentence**		**Fem**	**Masc**	**Fem**	**Masc**	**Fem**	**Masc**
**PAST TENSE**							
Noun Group	Unpaired	4914	4692	4258	3321	3884	3443
	Paired	4467	4567	4512	3612	4575	4017
	Colloquial	3748	4328	3877	3674	4479	4373
**PRESENT TENSE**							
Noun Group	Unpaired	4469	3628	3841	4321	3122	3358
	Paired	4018	3581	3455	3839	3715	4126
	Colloquial	3776	3914	4209	4008	4956	4229

*For the past tense passages, the gender of the pronoun in the second sentence is also the gender of the inflection of the verb in the first sentence*.

**Figure 2:**
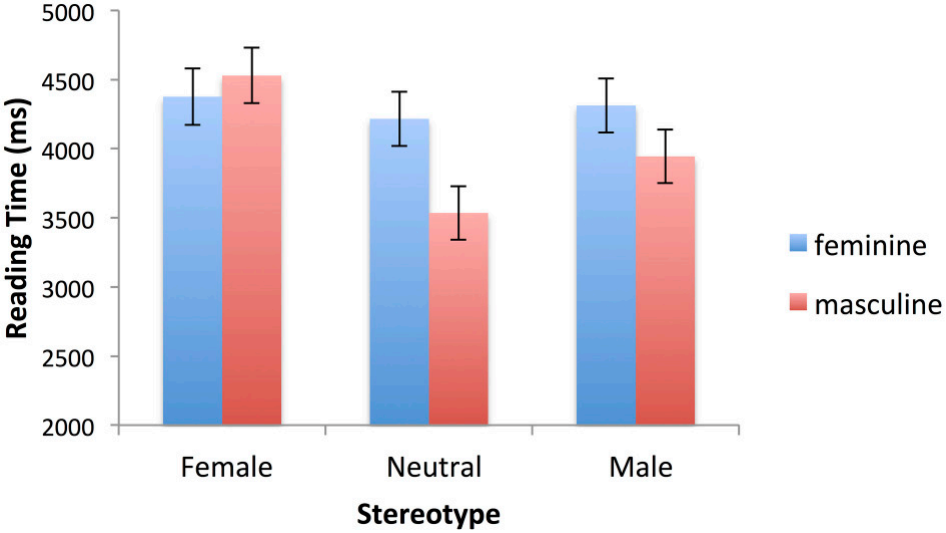
**Interaction of stereotype (female, neutral, male) and gender marking (masculine, feminine) in first sentence reading times (ms), for past tense specific sentences only**. Error bars represent standard errors calculated by the SPSS MIXED procedure.

For the first sentences there was a main effect of tense, *F*_(1, 1237.181)_ = 7.78, *p* = 0.005, Reading was faster for present tense passages than past tense passages (3920 vs. 4152 ms). For the past tense sentences only there was a main effect of the gender marking on the predicate *F*_(1, 573.102)_ = 3.691, *p* = 0.055., and a marginal interaction of gender marking and stereotype, *F*_(2, 597.831)_ = 2.739, *p* = 0.065. Reading times were higher when the gender marking was feminine (4301 vs. 4003 ms). In addition, times were higher when masculine gender marking mismatched a female stereotype than when it was consistent with a male or neutral stereotype. There were no differential effects of stereotype when the gender marking was feminine. The interaction pattern, including the stereotype match-mismatch effect can be seen in Figure 2. In the analysis of the residual reading times, the crucial 3-way interaction of tense, stereotype and gender marking was not significant, *F*_(2, 1253.111)_ = 1.709, *p* = 0.18), though it was significant in the analysis of the (trimmed) raw reading times, which we ran to compute the means reported in Table 4, *F*_(2, 1314)_ = 3.372, *p* = 0.035.

These results indicate that both grammatical and stereotypical gender information were used in the interpretation of the first sentences. In these sentences a masculine role noun was always used. However, both the sex of the person referred to, and the stereotype of the role could match with or clash with the grammatical gender of the noun (or bear a neutral relation in the case of the neutral stereotypes). When the person was female, the grammatical gender marking on the predicate of the first sentence mismatched the grammatical gender of the noun, when the sentence was in the past tense. In the present tense, the marking on the predicate was uninformative. So, in relation to the use of grammatical information, the main effect of tense on reading times for the first sentences showed that when there was more gender-related information to process (in the past tense), more time was taken to read the sentence, although the sentences did also differ in tense. Furthermore, in the past tense passages where it was clear in the first sentence whether the semantic gender of the intended referent matched or mismatched the grammatical gender of the subject noun, a clash with the grammatical gender of the subject noun slowed people down.

In relation to the use of stereotype information, in some cases the gender stereotype matched the grammatical gender of the role noun, and in others it mismatched. A standard stereotype mismatch effect would, therefore, have been reflected in a main effect of stereotype. In particular, male stereotype would be match and female mismatch. However, no evidence for such an effect was found. However, there was evidence that stereotype information was used in the processing of the first sentence, but its effects were only seen when it clashed with two pieces of grammatical gender information—a female stereotype plus the masculine noun and masculine gender marking on the verb slowed people down. In the present tense, when the stereotype clashed only with the masculine grammatical gender of the subject noun, no stereotype mismatch effect was found, so it appears that the stereotype effect is driven by the mismatch with grammatical inflections on the predicate.

**Figure 3:**
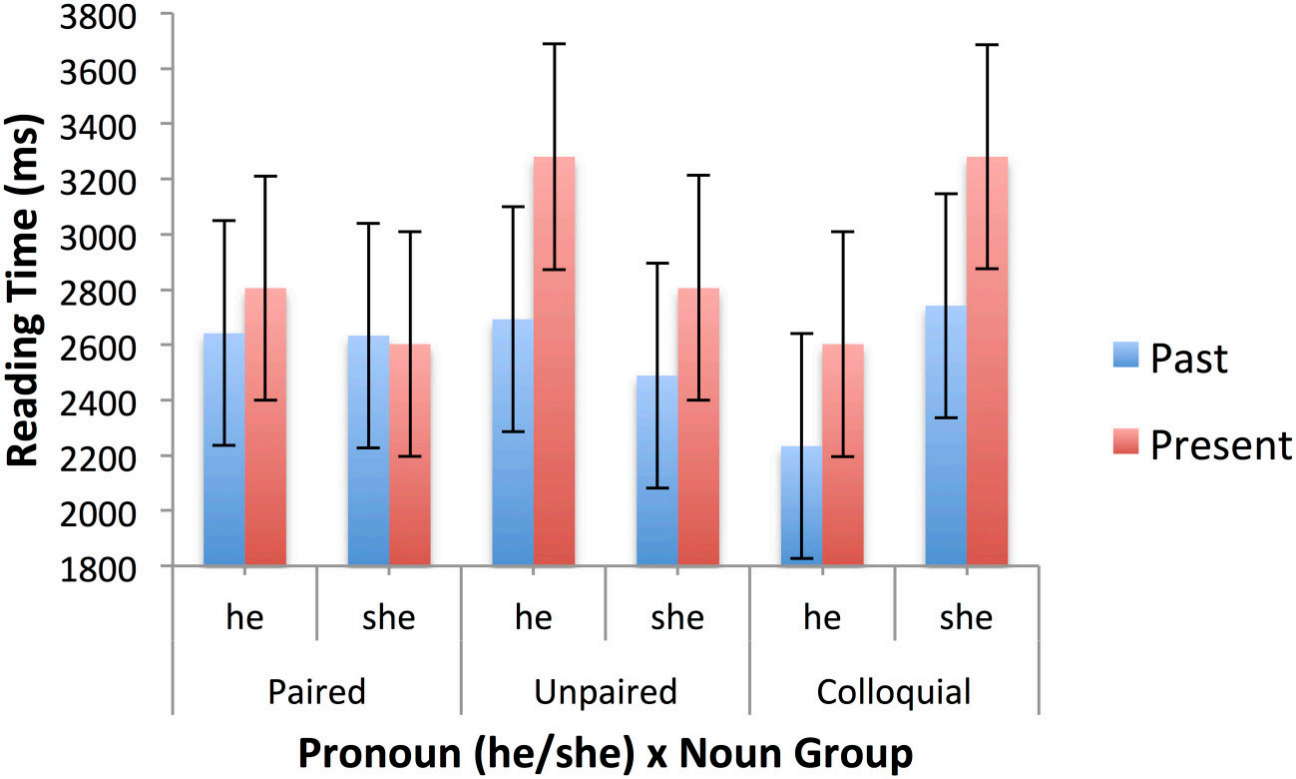
**Three-way interaction of tense (past, present), pronoun (she, he), and noun group (unpaired, paired, colloquial) in second sentence reading times (ms), for specific sentences**. Error bars represent standard errors calculated by the SPSS MIXED procedure.

**Figure 4:**
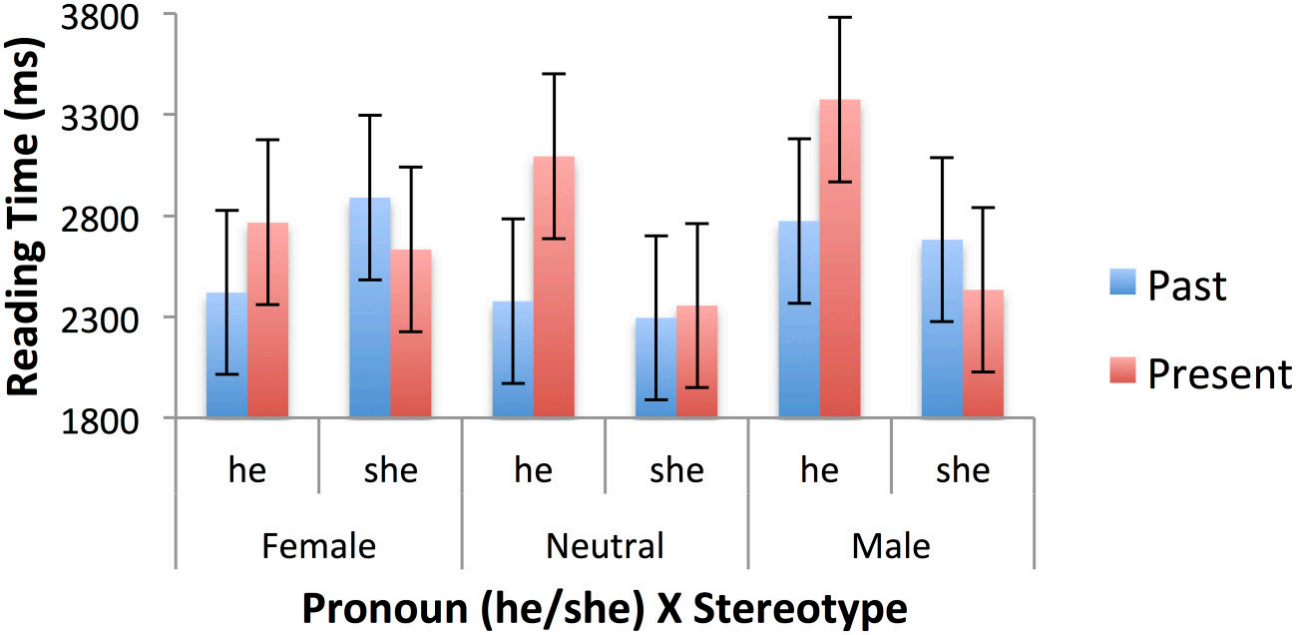
**Three-way interaction of tense (past, present), pronoun (she, he), and stereotype (female, neutral, male) in second sentence reading times (ms), for specific sentences**. Error bars represent standard errors calculated by the SPSS MIXED procedure.

Table 5 shows the mean raw reading times, after truncation, for the second sentences of the passages in the past and present tenses. Mixed models were fitted using the SPSS MIXED procedure, as for the first sentences, and with the same limitations (see footnote 2). There were main effects of tense, *F*_(1, 1282.039)_ = 7.111, *p* = 0.008, pronoun, *F*_(1, 1282.233)_ = 10.734, *p* = 0.002, and gender stereotype, *F*_(2, 62.080)_ = 2.609, *p* = 0.082. Responses were faster for passages with *he* rather than *she* (2548 vs. 2800 ms), for past tense passages than present tense passages (2572 vs. 2776 ms), and for passages with neutral stereotypes (2530 ms) than female (2677 ms) and male (2815 ms). There were also significant two-way interactions between tense and pronoun, *F*_(1, 1282.089)_ = 19.343, *p* < 0.001, and pronoun and stereotype, *F*_(2, 1295.076)_ = 5.605, *p* = 0.004). Finally, there was a significant three-way interaction of tense, pronoun and noun group, *F*_(2, 1284.519)_ = 3.126, *p* = 0.044. The pronoun effect was restricted to present tense passages 604 vs. –99 ms), and among those it occurred only for paired and colloquial nouns, not unpaired nouns, with the larger effect being for paired nouns. These effects are illustrated in Figure 3. The pronoun by stereotype interaction is clearly of interest to the literature on stereotypes. The pronoun “she” was read more slowly after male stereotyped nouns (3074 ms) than female (2592 ms.) or neutral (2735 ms), whereas “he” was read more quickly after male (2557 ms) and neutral (2325 ms) than female (2761 ms) stereotypes. Figure 4 shows these effects. In the past tense (blue bars), the standard stereotype match-mismatch effect is seen, including the usual advantage for masculine pronouns following neutral stereotypes, though this effect is numerically small in the current data set. The corresponding effect in the present tense (red bars) is overlaid on the main effect of pronoun, with sentences containing the masculine pronoun being read faster overall in the present tense.

**Table 5:** **Mean reading times (ms) for second sentences for the texts in which the nouns were intended to be interpreted specifically**.

**Stereotype**		**Female**		**Neutral**		**Male**	
**Pronoun**		**She**	**He**	**She**	**He**	**She**	**He**
**PAST TENSE**							
Noun Group	Unpaired	2535	2886	2474	2323	2918	2691
	Paired	2722	2828	2391	2060	2966	2578
	Colloquial	2001	2953	2264	2500	2436	2773
**PRESENT TENSE**							
Noun Group	Unpaired	2430	2595	2959	2666	3028	2547
	Paired	3081	2523	3052	2152	3710	2579
	Colloquial	2786	2780	3269	2248	3387	2172

Looking at the separate analyses of present and past tense passages, the pronoun effect was only significant in the present tense passages, *F*_(1, 599.227)_ = 31.677, *p* < 0.001. The pattern is consistent with the size of the effect in the two types of passage noted above. The pronoun by stereotype interaction was significant in the present tense passages, *F*_(2, 587.973)_ = 3.892, *p* = 0.021, and marginal in the past tense passages, *F*_(2, 626.234)_ = 2.53, *p* = 0.081. A pronoun by noun group interaction, which was marginal in the overall analysis, *F*_(2, 1286.932)_ = 2.724, *p* = 0.066, was significant in the present tense passages, *F*_(2, 612.097)_ = 3.408, *p* = 0.034, and marginal in the past tense passages, *F*_(2, 614.511)_ = 2.761, *p* = 0.064. When the pronoun is “she” there is more difficulty if the noun in the first sentence is paired and so has a feminine counterpart that was not used. This pattern is not seen for the pronoun “he.” Finally, for the past tense passages, there was a marginal effect of stereotype, *F*_(2, 76.619)_ = 2.828, *p* = 0.065. Neutral stereotypes led to faster reading of the second sentence in the past tense passages compared with all other conditions defined by stereotype x tense.

The pattern of reading times for the second sentences was complicated, and partly reflected differences in the information available in (and processed from) the first sentences. So, the effect of tense in the second sentence was the reverse of that in the first sentence. Given that more time had been devoted to the first sentences in the past tense passages (presumably because they contained more information), less was devoted to the second sentences in the same passages. Conversely, as the sex of the main character only became apparent in the second sentence of the present tense passages, more time was needed to process this information. As with the first sentences, there was evidence for the use of both grammatical and stereotypical information in the processing of the second sentences.

The initial pronoun in the second sentence carried the information about the actual sex of the character, which had also been indicated by the inflectional ending in the predicate in the first sentence of the past tense passages, but not of the present tense passages. In the present tense passages, therefore, any clash between the grammatical gender of the role name and the sex of the character, or between the stereotype of the role name and the sex of the character, could only become apparent in the second sentence. The effect of pronoun (“she” vs. “he”) in the second sentence, which was restricted to the present tense passages, reflects the match or mismatch between the pronoun and the grammatical gender of the role noun, and that fact that such a match or mismatch had already been observed in the first sentences of the past tense passages.

The pronoun by stereotype interaction in the second sentence is the standard stereotype match-mismatch effect reported elsewhere in the literature. There is some evidence, from the fact that the two-way interaction is only significant in the present tense, that the effect is stronger in the present tense passages, in which the second sentence is the first place in which the effect might be detected. In the past tense passages, the stereotype match-mismatch is present in the first sentence. However, the three-way interaction with tense is not significant and the pattern of the interaction is similar for past and present tense passages. In previous studies (e.g., Carreiras et al., 1996; Duffy and Keir, 2004), if such a clash appears early in a passage, it is dealt with at that point and does not affect later processing. The pattern is not so clear in the present study, which may be because inflections on verbs are less obvious indicator of a person's sex than the gender of a definite article (Carreiras et al., Spanish experiments) or the explicit use of the terms “male” and “female” (Duffy and Keir). One possibility is that, although inflections on past tense verbs are quite obvious gender indicators in spoken Russian, they may be less obvious in the written form as in most cases only a single character is added to a masculine form verb [*skazal—said (masc), skazal**a*—*said (fem)*]. However, such single character inflectional changes are not difficult to notice, at least when attention is drawn to them. More plausibly, it may be that, because of the proximity of the verb to the subject noun in our sentences, grammatical, rather than semantic agreement would be acceptable, so that the inflection on the verb is not a reliable indicator of the protagonist's sex.

Finally, the pronoun by noun group interaction is of some interest as it shows that readers are affected by other ways that a language makes available for expressing the same idea. For example, if a writer knows that a character is female and is going to indicate this fact by inflecting a past tense verb, it seems odd for that writer to use a masculine noun generically when a feminine counterpart is available (as in the case of paired nouns). However, the effect of this anomaly does not appear in first sentence reading times, again suggesting that the inflectional morphology on the predicate plays only a weak role in the representation of gender. Instead, it appears in the second sentence, where a personal pronoun provides more direct evidence of the person's sex.

#### Interpretation of generic nouns

##### Reading times

As for the specific items, examination of the histograms for the reading times suggested that 10 s was a sensible cut off to use for both first sentence and second sentence reading times. Again, as for the specific items, the lengths of the sentences showed considerable variability: first sentences mean = 68.2 characters, min = 39, max = 109; second sentences mean = 49.9, min = 29, max = 72. For the purposes of analysis, therefore, raw reading times were replaced with residual reading times, based on regressions of reading time against length in characters (including spaces) calculated separately for each participant and for each sentence (first and second).

Tables 6, 7 shows the mean raw reading times, after truncation, for the first and second sentences, respectively. Mixed effects models, with stereotype (female, neutral, male), continuation (women, men), and noun group (unpaired, paired, colloquial) as fixed effects were fitted to the residual reading time data using the SPSS MIXED procedure. A compound symmetry covariance structure was chosen for the repeated effects, and random intercepts (only, for the same reasons as stated in Footnote 2) were selected for both participants and materials, so no issue of covariance structure for random effects arose. All reported times are based on the actual set of times, after truncation, in the various conditions of the experiment.

**Table 6:** **Mean reading times (ms) for first sentences for the texts in which the nouns were intended to be interpreted generically**.

**Stereotype**		**Female**		**Neutral**		**Male**	
**Continuation**		**Women**	**Men**	**Women**	**Men**	**Women**	**Men**
Noun Group	Unpaired	5489	4359	6956	5329	4563	5412
	Paired	4743	4347	3711	3733	5546	6733
	Colloquial	4802	4858	4679	4917	4419	4912

**Table 7:** **Mean reading times (ms) for second sentences for the texts in which the nouns were intended to be interpreted generically**.

**Stereotype**		**Female**		**Neutral**		**Male**	
**Continuation**		**Women**	**Men**	**Women**	**Men**	**Women**	**Men**
Noun Group	Unpaired	3275	2412	3687	3026	3612	3293
	Paired	2647	2826	3365	3076	4935	3863
	Colloquial	3260	3233	3367	2622	3449	3292

No significant effects were found for the reading times of the first sentences.

For the second sentence, there was a marginal effect of continuation, *F*_(1, 661)_ = 3.721 *p* = 0.054, with slower reading times for “some of the women” (3511 ms) compared with “some of the men” (3071 ms). There was a also main effect of stereotype, *F*_(2, 661)_ = 3.016, *p* = 0.05, with reading times of 2942, 3190, and 3741 ms for female, neutral, and male stereotypes. However, the effect of stereotype in the reading times for the second sentence should be viewed with caution, even though we analyzed residual reading times, as it is a between-passages effect.

##### Judgements

Because of the possibility of non-generic interpretations, which would make *some of the women* infelicitous, we analyzed the judgment data for the “generic” passages. Table 8 shows the mean percentage of positive responses There was a main effect of continuation (“some of the women” vs. “some of the men”), *F*_(1, 658.489)_ = 12.178, *p* = 0.001, with more positive responses for men (79.3%) than for women (67.6%). There was also a two-way interaction between continuation and noun group, *F*_(2, 661.417)_ = 7.045, *p* = 0.001; and a three-way interaction of continuation, noun group, and stereotype, *F*_(4, 661.464)_ = 6.519, *p* < 0.001. Given the main effect of continuation, the two- and three-way interactions are primarily driven by the fact that “Some of the women” attracted a particularly low number of positive responses when the stereotype was male and the noun was from a paired couple. In this case, the use of the masculine noun, when an equivalent feminine noun is available, together with the male stereotype, reinforces the idea that the people being talked about are men.

**Table 8:** **Mean percentage of yes responses to questions for the texts in which the nouns were intended to be interpreted generically**.

**Stereotype**		**Female**		**Neutral**		**Male**	
**Continuation**		**Women**	**Men**	**Women**	**Men**	**Women**	**Men**
Noun Group	Unpaired	65.0	85.0	60.0	90.0	77.5	65.0
	Paired	75.0	70.0	68.0	82.0	30.0	90.0
	Colloquial	70.0	81.3	81.7	81.7	81.4	68.6

The effect of continuation, with “some of the men” continuations being read more quickly than “some of the women” continuations, and being accepted more frequently, parallels the findings of Gygax et al. (2008) for French and German. Those authors interpreted that finding as evidence that the masculine plural noun phrases (in the first sentences) were interpreted as referring to groups of males, rather than as generic references to group of both men and women or of unknown composition.

The interactions of continuation and noun group and continuation, noun group and stereotype in the judgments, despite including between-item comparisons, are relatively unproblematic, as judgment is not directly affected by length. It appears, as with the specific passages, that the use of the masculine member of a pair of nouns, when there is also a male stereotype, makes it particularly difficult to consider that there are women in the group.

## General discussion

We collected a set of stereotype norms for 160 Russian role names, and used a subset of these role names to construct short passages for an online study of the use of stereotype and other gender information in the interpretation of Russian. The stereotype norms showed a similar distribution to that found in other languages.

In the online study, Russian was of interest because of its morphological gender marking on (past tense) verbs, which typically agrees with the semantic gender of an animate referent, and also because of the existence of different (masculine) noun classes, which may or may not have corresponding feminine forms. When they do, the feminine forms may differ simply (or at least primarily) in referring to females rather than males. Or they may be “colloquial,” with their use restricted to the spoken language and often having derogatory connotations.

Our study explored similarities and differences between the processing of gender information in Russian and in other languages (English, French, German, Spanish) that we had previously studied. As in those other languages, we saw immediate deployment of gender information, both grammatical and stereotypical, in Russian. However, some differences did emerge, which could be related to properties of the Russian language. First, although there was evidence that people were sensitive to this information, grammatical gender marking on predicates (verbs) was not immediately and completely used to counteract stereotype information, unlike, for example, grammatical gender marking on Spanish definite articles (Carreiras et al., 1996). In Spanish *la futbolista* (the female footballer) was initially processed more slowly that *el futolista* (the male footballer). However, once the stereotype had been neutralized by the definite article (*la* vs. *el*) it was just as easy to refer to the female footballer with a feminine pronoun as to refer to the male footballer with a masculine pronoun. In our Russian experiment, the detection of a mismatch between a stereotype and the actual sex of the person referred to did not result in the mismatch being completely resolved, so that it did affect later processing.

Second, there was also evidence that the different classes of noun, which do not have direct counterparts in the other languages we have studied, behaved in different ways. In particular, when a masculine noun has a straightforward feminine counterpart, the oddity of using the masculine form when later information in the sentence suggests that the writer knows the person is female causes additional processing difficulty.

Our findings, therefore, show that even in a language like Russian, which has a more complex noun class and gender system than other languages previously studied, gender information is processed quickly and easily to provide detailed representations of the characters described in a text. However, the complexities of Russian do result in effects that were not seen in other languages, effects that can be clearly related to properties of the Russian language.

### Conflict of interest statement

The authors declare that the research was conducted in the absence of any commercial or financial relationships that could be construed as a potential conflict of interest.

## Footnotes

^1^All transliterations in this paper follow the BGN/PCGN system.

^2^We used four electronic dictionaries to allocate nouns to the three noun groups. T.F. Yefremova. The Comprehensive Dictionary of the Contemporary Russian Language. ©2006; http://www.lingoes.net/en/dictionary/dict_down.php?id=79A76AF19099A343BD990EC195719601. S.A. Kuznetsov. Comprehensive Russian Explanatory Dictionary ©2010; http://www.lingvo-online.ru/en/LingvoDictionaries/Details?dictionary=ExplanatoryBTS%20%28Ru-Ru%29. Dmitry Ushakov. Explanatory dictionary of Russian. ©≪ ACT, Astrel≫, Moscow, 2000; http://dic.academic.ru/contents.nsf/ushakov/. S. Ozhegov and N. Shvedova. Explanatory dictionary of Russian. ©Very Ltd., Israel, 1994. http://dic.academic.ru/contents.nsf/ogegova/. We checked if each masculine noun had a feminine form. If it did not it was added to the unpaired class. If the feminine noun was labeled *razg*. (colloquial), *ustar*. (archaic) or *prost*. (vulgar) the masculine noun was added to colloquial class. The remaining nouns formed the paired class.

^3^Psychology Software Tools Inc. [E.-Prime 2.0]. (2012). Retrieved from http://www.pstnet.com.

^4^Ideally, we would have fitted a full model, with random intercepts for all repeated effects, as recommended by Barr et al. (2013). However, neither SPSS MIXED nor lmer from the R package lme4 would converge on such a model a reasonable time (several days).
