# Supplementary material for: The Interaction of Morphological and Stereotypical Gender Information in Russian
Source: Front Psychol. 2015 Nov 16;6:1720. doi: 10.3389/fpsyg.2015.01720 (PMC4644804; doi:10.3389/fpsyg.2015.01720)
Supplement: Supplementary file 1 [file DataSheet1.PDF]

Appendix A. Online questionnaire design. Role nouns were presented only in the masculine plural form, as generic, on the left side. The 11-point rating scale was oriented towards 100 % *men* (from 100 % *women* and 0 % *men* to 0 % *women* and 100 % *men*) as depicted bellow. The English translations of the role nouns are shown here, but were not included in the study itself.

1. Пожалуйста, оцените соотношение (пропорцию) женщин и мужчин в предложенных ниже социальных группах.

		Женщины / Мужчины										
		100/0	90/10	80/20	70/30	60/40	50/50	40/60	30/70	20/80	10/90	0/100
**a.** Флористы	(Florists)	○	○	○	○	○	○	○	○	○	○	○
**b.** Баскетболисты	(Basketball players)	○	○	○	○	○	○	○	○	○	○	○
**c.** Водители	(Drivers)	○	○	○	○	○	○	○	○	○	○	○
**d.** Парикмахеры	(Hairdressers)	○	○	○	○	○	○	○	○	○	○	○
**e.** Председатели	(Chairmen)	○	○	○	○	○	○	○	○	○	○	○
**f.** Пианисты	(Piano players)	○	○	○	○	○	○	○	○	○	○	○
**g.** Продавцы	(Salespersons)	○	○	○	○	○	○	○	○	○	○	○
**h.** Юристы	(Jurists)	○	○	○	○	○	○	○	○	○	○	○
**i.** Певцы	(Singers)	○	○	○	○	○	○	○	○	○	○	○
**j.** Модельеры	(Dress designers)	○	○	○	○	○	○	○	○	○	○	○
**k.** Космонавты	(Astronauts)	○	○	○	○	○	○	○	○	○	○	○
**l.** Диджеи	(Disk jockeys)	○	○	○	○	○	○	○	○	○	○	○
**m.** Танцоры	(Dancers)	○	○	○	○	○	○	○	○	○	○	○
**n.** Скульпторы	(Sculptors)	○	○	○	○	○	○	○	○	○	○	○
**o.** Клоуны	(Clowns)	○	○	○	○	○	○	○	○	○	○	○
**p.** Официанты	(Waiters)	○	○	○	○	○	○	○	○	○	○	○
**q.** Массажисты	(Massage therapists)	○	○	○	○	○	○	○	○	○	○	○
**r.** Гитаристы	(Guitarists)	○	○	○	○	○	○	○	○	○	○	○
**s.** Акробаты	(Acrobats)	○	○	○	○	○	○	○	○	○	○	○
**t.** Преподаватели	(Lecturers)	○	○	○	○	○	○	○	○	○	○	○

Appendix B.

List of role nouns with mean ratings and standard deviations (scale midpoint = 6). Nouns that were used in the reading experiment marked * (singular) or ** (plural).

Role noun in Russian (read phonetically)	Role noun in English	Personal noun group	Mean rating (SD) by all respondents (N=106)	Mean rating (SD) by female respondents (N=87)	Mean rating (SD) by male respondents (N=16)
** Bibliotekari	Librarians	colloquial	2.3 (1.1)	2.3 (1.2)	2.2 (1.1)
* Kosmetologi	Beauticians	unpaired	2.3 (1.1)	2.3 (1.1)	2.3 (0.8)
** Vospitateli	Kindergarten teachers	paired	2.3 (1.3)	2.2 (1.3)	2.6 (1.5)
* Sekretari	Secretaries	colloquial	2.6 (1.4)	2.5 (1.4)	2.9 (1.4)
* Floristy	Florists	unpaired	2.7 (1.7)	2.6 (1.8)	2.8 (1.4)
* SHkol'nyye psikhologi	School psychologists	unpaired	2.7 (1.4)	2.7 (1.4)	3.0 (1.3)
** Kassiry	Cashiers	colloquial	3.0 (1.5)	3.0 (1.5)	2.6 (1.2)
* Bufetchiki	Bartenders	paired	3.1 (1.6)	3.1 (1.6)	3.3 (1.5)
* Biletery (v kinoteatrakh)	Ticket collectors	colloquial	3.2 (1.7)	3.1 (1.7)	3.4 (1.5)
Vizazhisty	Make up artists	colloquial	3.5 (1.7)	3.5 (1.7)	3.1 (1.6)
** Sotsial'nyye rabotniki	Social workers	colloquial	3.6 (1.4)	3.5 (1.4)	3.8 (1.4)
** Instruktora po aerobike	Aerobics instructors	colloquial	3.6 (1.6)	3.5 (1.5)	4.1 (1.6)
* Pediatry	Pediatricians	unpaired	3.6 (1.7)	3.5 (1.6)	4.3 (1.8)
** Filologi	Philologists	unpaired	3.7 (1.7)	3.7 (1.7)	3.3 (1.3)
* Uchitelya	School teachers	paired	3.7 (1.4)	3.6 (1.5)	3.9 (1.3)
** Plovtsy sinkhronnogo plavaniya	Synchronized swimmers	colloquial	3.7 (2.5)	3.8 (2.4)	3.3 (2.8)
* Akushery	Obstetricians	paired	3.8 (1.6)	3.9 (1.7)	3.1 (1.4)
* Administratory (v gostinitsakh)	Receptionists	colloquial	3.8 (1.7)	3.7 (1.6)	3.8 (2.4)
* Bukhgaltery	Accountants	colloquial	3.8 (1.3)	3.9 (1.3)	3.3 (1.0)
** Komendanty (v obshchezhitiyakh)	Hostel wardens	colloquial	4.0 (2.1)	4.1 (2.2)	3.5 (1.8)
* Bortprovodniki	Air stewards	paired	4.0 (2.1)	4.1 (2.1)	3.3 (1.3)
* Pedagogi	Pedagogues	unpaired	4.0 (1.3)	4.1 (1.3)	3.7 (1.1)
* Diyetologi	Dieticians	unpaired	4.0 (1.7)	4.1 (1.7)	3.8 (1.7)
* Instruktora po sheypingu	Instructors in shaping	colloquial	4.1 (1.9)	4.0 (1.9)	4.6 (2.0)
* Tovarovedy	Goods managers	unpaired	4.1 (1.7)	4.1 (1.8)	3.9 (1.4)
Konduktory (tramvayev, avtobusov)	Ticket inspector (in the bus)	colloquial	4.2 (2.5)	4.1 (2.4)	4.2 (2.1)
* Ekskursovody	Tour guides	colloquial	4.2 (1.5)	4.3 (1.5)	3.9 (1.1)
* Parikmakhery	Hairdressers	colloquial	4.3 (1.7)	4.4 (1.8)	3.7 (1.0)
** Dizaynery po inter'yeram	Interior designers	unpaired	4.3 (1.4)	4.4 (1.4)	3.9 (1.3)
* Striptizëry	Strippers	paired	4.3 (2.1)	4.3 (2.0)	4.5 (2.2)
* Farmatsevty	Pharmacists	unpaired	4.3 (1.5)	4.3 (1.6)	4.1 (1.2)
** Prepodavateli	Lecturers	paired	4.4 (1.5)	4.4 (1.5)	4.1 (1.6)
Korrektory	Proofreaders	colloquial	4.4 (1.9)	4.4 (1.9)	4.1 (1.7)
* Prodavtsy	Salespersons	paired	4.4 (1.5)	4.4 (1.5)	4.5 (1.9)
* Konsul'tant-prodavtsy	Consultant sellers	paired	4.6 (1.6)	4.5 (1.5)	4.8 (1.8)
* Konditery	Candy makers	colloquial	4.6 (1.6)	4.5 (1.6)	4.6 (1.5)
* Laboranty	Laboratory assistants	paired	4.7 (1.6)	4.7 (1.7)	4.4 (1.7)
Literaturovedy	Literary critics	unpaired	4.7 (1.6)	4.7 (1.7)	4.5 (1.4)
Gidy	Guides	unpaired	4.8 (1.6)	4.8 (1.5)	4.9 (2.1)
Dispetchery	Traffic controllers	unpaired	4.8 (2.1)	4.8 (2.2)	4.7 (2.0)
Psikhologi	Psychologists	unpaired	5.0 (1.5)	5.0 (1.5)	4.9 (1.7)
Artisty baleta	Ballet dancers	paired	5.0 (1.3)	4.9 (1.2)	5.6 (1.2)
Pochtal'ony	Mailmans	colloquial	5.1 (2.2)	5.2 (2.2)	4.3 (2.0)
Ofitsianty	Waiters	paired	5.2 (1.3)	5.2 (1.2)	5.3 (1.5)
Notariusy	Notary publics	unpaired	5.4 (1.8)	5.4 (1.8)	4.9 (2.0)
CHitateli	Readers	paired	5.4 (1.0)	5.4 (1.0)	5.7 (1.0)
KHoreografy	Choreographers	unpaired	5.5 (1.4)	5.5 (1.5)	5.5 (1.4)
Voditeli tramvayev	Tram drivers	colloquial	5.5 (2.4)	5.7 (2.5)	4.3 (1.9)
Peshekhody	Pedestrians	unpaired	5.6 (1.0)	5.6 (0.9)	5.4 (1.6)
Tantsory	Dancers	colloquial	5.6 (1.3)	5.6 (1.4)	5.3 (1.2)
Pekari	Bakers	colloquial	5.6 (1.5)	5.6 (1.5)	5.8 (1.5)
* Vrachi	Doctors	colloquial	5.6 (1.0)	5.7 (1.0)	5.4 (1.1)
Figuristy	Figure skaters	paired	5.6 (0.9)	5.7 (0.9)	5.4 (1.0)
* Massazhisty	Massage therapists	paired	5.7 (1.7)	5.8 (1.7)	5.6 (1.8)
** Studenty	Students	paired	5.7 (0.7)	5.6 (0.8)	5.9 (0.3)
* Povara	Cooks	colloquial	5.7 (1.8)	5.7 (1.8)	5.9 (1.9)
* Pevtsy	Singers	paired	5.8 (1.0)	5.8 (1.0)	5.8 (0.9)
* Gimnasty	Gymnasts	paired	5.8 (1.1)	5.8 (1.2)	5.9 (0.9)
* Redaktory	Editors	colloquial	5.8 (1.5)	5.8 (1.4)	5.6 (1.8)
* Stomatologi	Stomatologists	unpaired	5.8 (1.1)	5.9 (1.2)	5.6 (0.8)

** Aktery	Actors	paired	5.9 (0.6)	5.8 (0.6)	5.9 (0.7)
* Fiziologi	Physiologists	unpaired	5.9 (1.6)	5.9 (1.6)	5.6 (1.9)
* Dantisty	Dentists	paired	5.9 (1.5)	6.0 (1.5)	5.3 (1.5)
* Marketologi	Marketers	colloquial	5.9 (1.5)	5.9 (1.4)	5.8 (2.1)
* Doktora	Doctors	colloquial	5.9 (1.3)	5.9 (1.3)	6.1 (1.5)
** Strakhovyye agenty	Insurance agents	colloquial	6.0 (1.7)	6.0 (1.6)	6.0 (2.0)
* Nevrologi	Neurologists	unpaired	6.0 (1.5)	6.1 (1.4)	5.5 (2.0)
* Artisty	Artists	paired	6.0 (0.8)	6.0 (0.9)	6.0 (0.6)
** ZHurnalisty	Journalists	paired	6.0 (1.1)	6.1 (1.0)	5.9 (1.3)
** Agenty po prodazhe nedvizhimosti	Real estate agents	colloquial	6.1 (1.6)	6.1 (1.7)	6.3 (1.3)
* Veterinary	Veterinarians	colloquial	6.1 (1.5)	6.2 (1.5)	5.6 (2.0)
** Tennisisty	Tennis players	paired	6.1 (1.3)	6.1 (1.4)	6.3 (0.6)
* Malyary	House-painters	colloquial	6.2 (1.9)	6.3 (1.8)	4.9 (1.9)
** Ekonomisty	Economists	colloquial	6.2 (1.5)	6.2 (1.5)	6.3 (1.8)
** Zoologi	Zoologists	unpaired	6.2 (1.5)	6.3 (1.4)	5.6 (1.6)
* Illyustratory	Illustrators	unpaired	6.2 (1.4)	6.2 (1.4)	6.1 (1.4)
** Muzykanty	Musicians	colloquial	6.3 (1.0)	6.2 (1.0)	6.6 (1.0)
* Narodnyye artisty	People's Artists	paired	6.4 (0.8)	6.4 (0.7)	6.6 (1.4)
* Sportsmeny	Sportsmans	paired	6.4 (1.0)	6.4 (1.0)	6.4 (0.8)
** Aspiranty	Postgraduates	paired	6.4 (1.3)	6.4 (1.1)	6.1 (1.6)
* Menedzhery	Managers	unpaired	6.4 (1.1)	6.4 (1.2)	6.6 (1.0)
* Diktory radio	Radio announcers	colloquial	6.4 (1.4)	6.5 (1.4)	6.3 (1.3)
* Korrespondenty	Correspondents	paired	6.4 (1.1)	6.5 (1.1)	6.4 (1.0)
* Meteorologi	Meteorologists	unpaired	6.4 (1.4)	6.6 (1.4)	5.6 (1.4)
* Psikhiatry	Psychiatrists	unpaired	6.5 (1.7)	6.4 (1.6)	6.9 (1.5)
* Dvorniki	Street cleaners	colloquial	6.5 (1.9)	6.6 (1.8)	6.4 (2.5)
* Igloterapevty	Acupuncturists	unpaired	6.6 (1.9)	6.5 (1.9)	6.9 (2.1)
** Avtory	Authors	colloquial	6.6 (1.1)	6.6 (1.1)	6.8 (1.4)
** Model'yery	Dress designers	colloquial	6.6 (1.6)	6.6 (1.7)	6.4 (1.3)
Lyzhniki	Skiers	paired	6.6 (1.1)	6.6 (1.1)	6.7 (1.0)
Akrobaty	Acrobats	paired	6.7 (1.6)	6.8 (1.6)	6.4 (1.7)
Mastera sporta	Masters of sports	unpaired	6.7 (1.1)	6.7 (1.1)	6.4 (0.9)
Velosipedisty	Bikers	paired	6.7 (1.1)	6.7 (1.2)	6.6 (1.1)
Plovtsy	Swimmers	colloquial	6.7 (1.0)	6.7 (1.0)	6.8 (1.1)
Pianisty	Piano players	paired	6.8 (1.5)	6.8 (1.5)	7.0 (1.6)
Fotografy	Photographers	unpaired	6.8 (1.3)	6.9 (1.3)	6.6 (1.2)
Trenery	Coaches	unpaired	6.8 (1.2)	6.9 (1.3)	6.7 (1.1)
YUristy	Jurists	colloquial	6.9 (1.3)	6.9 (1.3)	6.9 (1.4)
KHudozhniki	Painters	paired	6.9 (1.3)	6.9 (1.4)	7.1 (1.4)
Poety	Poets	paired	6.9 (1.3)	6.9 (1.4)	6.7 (1.3)
Sud'i	Judges	unpaired	6.9 (1.7)	7.1 (1.7)	6.2 (2.1)
Pisateli	Writers	paired	6.9 (1.3)	6.9 (1.2)	7.1 (1.4)
Advokaty	Lawyers	unpaired	7.1 (1.3)	7.2 (1.3)	7.0 (1.4)
Finansisty	Financial experts	unpaired	7.3 (1.6)	7.3 (1.5)	7.4 (2.1)
Delegaty	Delegates	colloquial	7.3 (1.7)	7.4 (1.8)	7.1 (1.5)
Inzhenery	Engineers	colloquial	7.3 (1.2)	7.3 (1.2)	7.8 (1.4)
Fekhtoval'shchiki	Fencers	paired	7.3 (1.5)	7.4 (1.5)	6.6 (1.7)
Dotsenty	Associate professors	unpaired	7.4 (1.4)	7.4 (1.4)	7.1 (1.4)
Arkhitektory	Architects	unpaired	7.4 (1.7)	7.5 (1.7)	7.5 (2.0)
* Basketbolisty	Basketball players	paired	7.5 (1.8)	7.5 (1.8)	7.6 (1.9)
* Komedianty	Comedians	paired	7.5 (1.4)	7.5 (1.4)	7.4 (1.5)
* Uchënyye	Scientists	unpaired	7.5 (1.4)	7.4 (1.4)	7.9 (1.6)
Issledovateli	Explorers	colloquial	7.5 (1.8)	7.6 (1.6)	6.9 (2.4)
* Predprinimateli	Entrepreneurs	paired	7.6 (1.3)	7.6 (1.2)	7.4 (1.5)
Atlety	Athletes	unpaired	7.7 (1.5)	7.6 (1.6)	8.4 (1.4)
** Fermery	Farmers	colloquial	7.7 (1.5)	7.7 (1.5)	7.6 (1.4)
Dramaturgi	Dramatists	unpaired	7.7 (1.5)	7.7 (1.5)	7.6 (1.3)
Predsedateli	Chairmans	colloquial	7.8 (2.1)	7.9 (2.0)	7.4 (2.4)
* Stsenaristy	Screenwriters	paired	7.8 (1.2)	7.9 (1.1)	7.3 (1.7)
SHpiony	Spies	paired	7.9 (1.7)	7.9 (1.7)	7.4 (1.5)
** Direktora	Directors	colloquial	7.9 (1.4)	7.9 (1.4)	8.0 (1.2)
* Professora	Professors	colloquial	8.0 (1.4)	8.0 (1.3)	7.9 (1.7)
ZHonglëry	Jugglers	colloquial	8.0 (1.6)	8.1 (1.6)	7.7 (1.7)

** Voditeli	Drivers	colloquial	8.0 (1.9)	8.0 (1.9)	7.9 (2.1)
* Skul’ptory	Sculptors	colloquial	8.2 (1.5)	8.2 (1.5)	8.1 (1.7)
* Prokurory	Attorneys	colloquial	8.2 (1.7)	8.3 (1.6)	7.8 (2.1)
** Akvalangisty	Scuba divers	paired	8.2 (1.5)	8.3 (1.5)	8.0 (1.6)
* Gitaristy	Guitarists	paired	8.3 (1.6)	8.3 (1.5)	7.9 (2.1)
* Matematiki	Mathematicians	colloquial	8.3 (1.5)	8.1 (1.6)	9.1 (1.2)
** Komiki	Comedians	unpaired	8.3 (1.5)	8.3 (1.5)	8.3 (1.4)
* Torgovtsy antikvariata	Antique dealers	colloquial	8.3 (1.6)	8.2 (1.6)	8.6 (1.3)
* Kommentatory	Commentators	colloquial	8.3 (1.4)	8.2 (1.4)	8.6 (1.2)
* Didzhei	Disk jockeys	unpaired	8.4 (1.7)	8.4 (1.8)	8.3 (1.3)
* Kompozitory	Composers	colloquial	8.4 (1.3)	8.4 (1.3)	8.5 (1.4)
** KHirurgi	Surgeons	unpaired	8.4 (1.2)	8.4 (1.2)	8.4 (1.4)
** Sledovateli	Detectives	colloquial	8.4 (1.3)	8.5 (1.2)	8.1 (1.2)
Kinorezhissëry	Movie directors	unpaired	8.5 (1.2)	8.4 (1.3)	9.0 (0.8)
** Akademiki	Academicians	unpaired	8.5 (1.5)	8.5 (1.5)	8.6 (1.8)
* Klouny	Clowns	unpaired	8.6 (1.6)	8.7 (1.6)	8.3 (1.5)
* Filosofy	Philosophers	unpaired	8.6 (1.7)	8.5 (1.7)	8.8 (1.7)
* Deputaty	Deputys	colloquial	8.6 (1.2)	8.6 (1.2)	8.3 (1.1)
** Izobretateli	Inventors	colloquial	8.6 (1.5)	8.6 (1.5)	8.8 (1.3)
* Politiki	Politicians	unpaired	8.8 (1.2)	8.8 (1.2)	8.7 (0.9)
* Programmisty	Computer programmers	unpaired	8.9 (1.1)	8.9 (1.1)	8.8 (1.3)
** Prezidenty kompaniy	Company presidents	colloquial	8.9 (1.5)	8.9 (1.5)	9.0 (1.8)
* Kinomekhaniki	Projectionists	unpaired	9.2 (1.2)	9.2 (1.2)	9.3 (1.0)
Storozha	Watchmans	colloquial	9.3 (1.5)	9.4 (1.3)	8.6 (2.0)
* Kosmonavty	Astronauts	colloquial	9.4 (1.3)	9.4 (1.3)	9.3 (0.9)
* Futbolisty	Footballers	paired	9.5 (1.3)	9.5 (1.1)	9.1 (2.1)
Myasniki	Butchers	unpaired	9.7 (1.3)	9.7 (1.3)	9.8 (0.8)
Okhranniki	Guards	colloquial	9.7 (1.5)	9.6 (1.5)	9.9 (1.2)
Piloty	Pilots	unpaired	9.7 (1.1)	9.8 (1.0)	9.8 (0.9)
Gubernatory	Governors	colloquial	9.7 (1.0)	9.7 (1.0)	9.8 (0.7)
Boksëry	Boxers	unpaired	9.8 (1.1)	9.8 (1.1)	9.9 (0.6)
Plotniki	Carpenters	unpaired	9.8 (1.6)	10 (1.3)	8.8 (2.5)
Avtomekhaniki	Auto mechanics	unpaired	9.8 (1.4)	9.8 (1.4)	9.7 (1.2)
Polkovniki	Colonels	unpaired	9.9 (1.1)	9.9 (1.1)	10.1 (0.6)
SHveytsary	Doorkeepers	unpaired	9.9 (1.6)	10.0 (1.7)	9.9 (1.1)
Pozharniki	Fire fighters	unpaired	10.2 (1.0)	10.2 (1.0)	10.2 (0.7)
Admiraly	Admirals	unpaired	10.6 (0.8)	10.7 (0.7)	10.8 (0.5)
